# Surviving a Rare Case: Successful Endovascular Treatment for Multiple Dural Venous Sinus Thrombosis With Dural Arteriovenous Fistula

**DOI:** 10.7759/cureus.55238

**Published:** 2024-02-29

**Authors:** Harshitha Reddy, Faizanulla Khan, Sunil Kumar, Sourya Acharya, Pankaj Banode, Rahul Desale

**Affiliations:** 1 Internal Medicine, Jawaharlal Nehru Medical College, Datta Meghe Institute of Higher Education & Research, Wardha, IND; 2 Interventional Radiology, Jawaharlal Nehru Medical College, Datta Meghe Institute of Higher Education & Research, Wardha, IND

**Keywords:** thrombosis, vein, artery, embolisation, fistula

## Abstract

Vascular anomalies known as dural arteriovenous fistulas (DAVFs) occur when arteries that emerge from carotid or vertebral artery branches empty straight into the dural venous sinuses. A 16-year-old male patient at the center of this case study initially arrived at the hospital with symptoms of generalized tonic-clonic seizures and headaches accompanied by vomiting, followed by right-sided hemiparesis and subsequently left-sided hemiparesis. An MRI brain with MR angiography was performed, revealing an abnormal fistulous connection between the sigmoid and transverse sinus and the branches of the bilateral external carotid and right internal carotid artery. Embolization was performed using a mixture of glue and lipoid to address the issue.

## Introduction

Within the brain, atypical connections between arteries and veins are referred to as dural arteriovenous fistulas (DAVFs). Intracranial DAVFs are abnormal direct shunts between the meningeal veins, cortical veins, or dural arteries, and the dural venous sinuses [1]. The most often documented sites for DAVFs in adult patients are the cavernous sinus and the transverse-sigmoid sinus region, with a left-sided predominance. Typically, DAVFs are situated supratentorially rather than infratentorially. They can also be found in the Sylvian, tentorial, petrosal, ethmoidal, cavernous, spinal dura, and superior sagittal sinuses [2]. These anomalies are situated either inside or near the dural sinus wall, constituting 10-15% of all cerebral arteriovenous malformations. There is a 0.15-0.29 annual incidence of DAVFs per 100,000 people. The occurrence of DAVFs varies according to location, with 50% found in the transverse sinus, 16% in the cavernous sinus, 12% in the tentorium, and 2% in the anterior fossa [2].

Most instances of these abnormalities are caused by prior neurosurgical interventions, confined infections, intracranial or spinal tumors, head injuries, and hypercoagulation disorders. Although uncommon, these anomalies can give rise to diverse neurological symptoms, spanning from mild manifestations to profound neurological impairments. The clinical characteristics of DAVFs are mainly influenced by their venous drainage patterns, dictating the potential for intracranial hypertension and bleeding. To circumvent complications associated with venous congestion and hemorrhage, prompt identification and intervention are imperative [3].

## Case presentation

A 16-year-old male was brought to the casualty with complaints of a throbbing headache followed by vomiting for one week, which was of insidious onset, gradually progressive, throbbing type more in the parietal and temporal region, which aggravated the cough. The patient also developed weakness of the right lower limb, which was sudden in onset and gradually progressed to the right upper limb and then to the left side with one episode of generalized tonic-clonic seizure before hospital admission. The patient did not have blurring of vision, trauma, or deviation of the angle of the mouth. The patient was put on a mechanical ventilator as his Glasgow Coma Scale was 4/15.

The blood pressure of the patient on admission was 130/80, and the pulse rate was 60 bpm with a respiratory rate of 28 per minute. On the CNS examination, his pupillary reflex was absent, and the bilateral plantar was extensor. Other systemic examinations were normal. The patient was evaluated for papilledema and was normal on fundoscopy. His routine laboratory parameters have been highlighted in Table 1.

**Table 1:** Laboratory parameters of the patient

Laboratory investigations	Value in the patient	Biological reference range
Hemoglobin	11	13-15 g/dl
Total leucocyte count	10,700	4,000-11,000/cumm
Platelet count	229,000	150,000-450,000/cumm
Mean corpuscular volume	84	79-100 fl
Urea	21	9-20 mg/dl
Creatinine	1.1	0.6-1.2 mg/dl
Sodium	139	137-144 mmol/l
Potassium	4.1	3.5-5.1 mmol/l
Alkaline phosphatase	121	38-126 U/l
Alanine transaminase	19	<50 U/l
Aspartate transaminase	22	17-59 U/l
Total protein	6.4	6.3-8.2 gm/dl
Albumin	3.8	3.5-5 gm/dl
Total bilirubin	0.6	0.2-1.3 mg/dl
Conjugated bilirubin	0.2	0-0.3 mg/dl
Unconjugated bilirubin	0.4	0-1.1 mg/dl
Globulin	2.6	2.3-3.5 gm/dl

**Figure 1:**
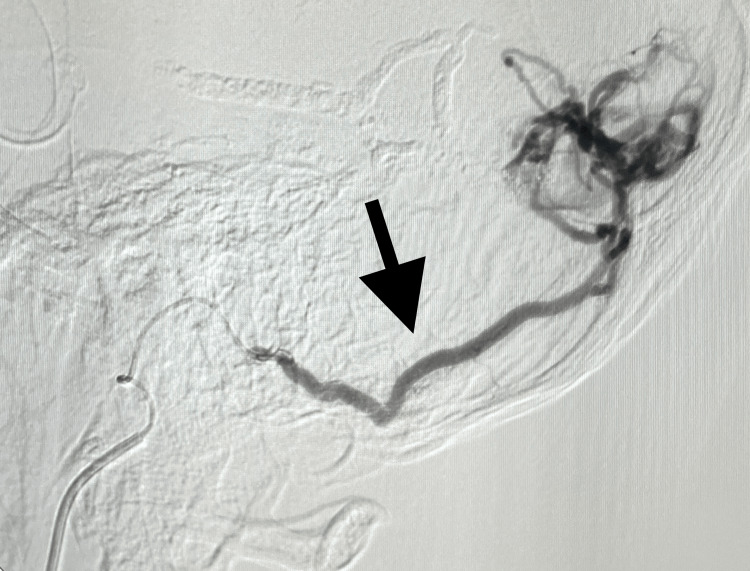
Connection between the external carotid and transverse sinus before applying the onyx glue (black arrow)

**Figure 2:**
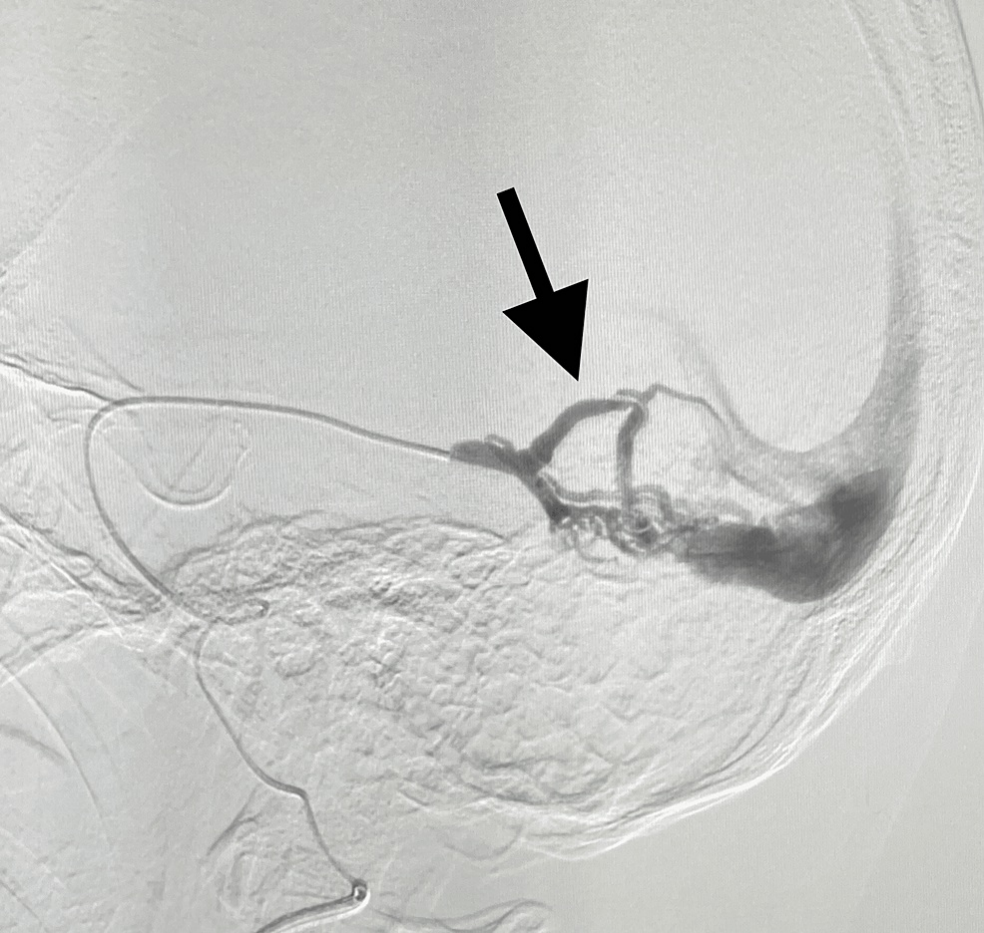
Arteriovenous fistula connecting the internal carotid and the cavernous sinus visualized with dye (black arrow)

His MR angiography was done, which showed multiple DAVFs between the sigmoid and transverse sinus and the branches of the bilateral external carotid and right internal carotid arteries with retrograde flow, respectively, as shown in Figure 1 and Figure 2.

**Figure 3:**
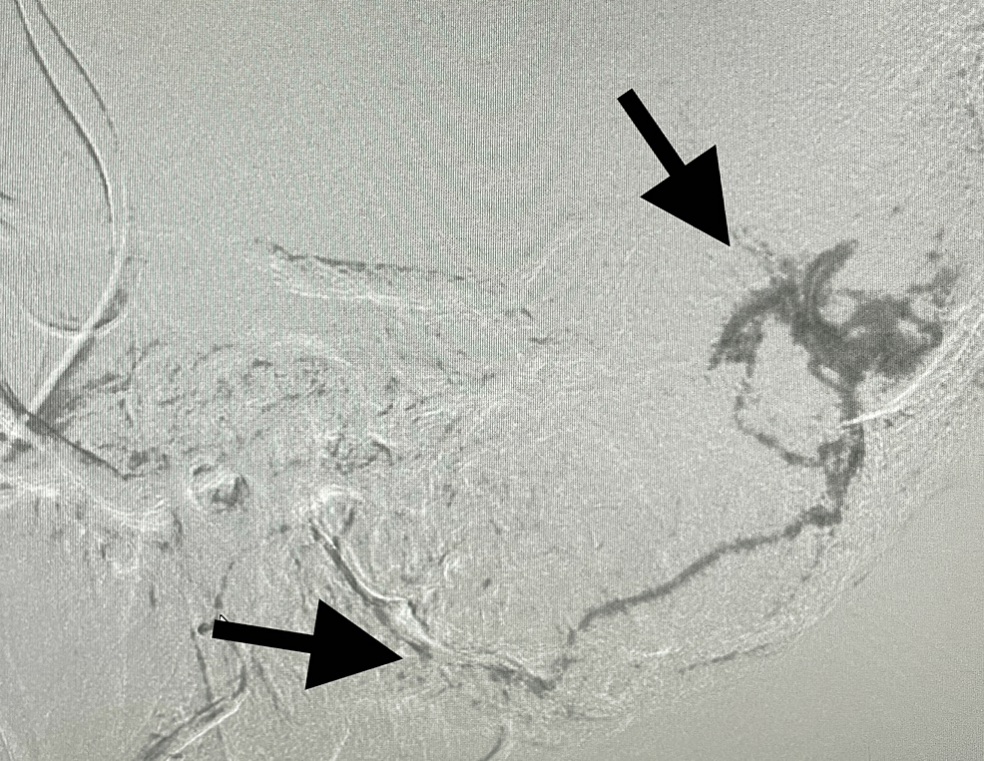
Closure of the connection between the external carotid and the transverse sinus after glue placement (black arrow)

The patient was started on injectable mannitol and injectable levetiracetam. Transarterial glue embolization therapy was performed in light of the abnormal venous connection between the external carotid artery, the internal carotid artery, and the dural sinuses. The closure of the connection between the external carotid and the transverse sinus after glue placement is shown in Figure 3.

**Figure 4:**
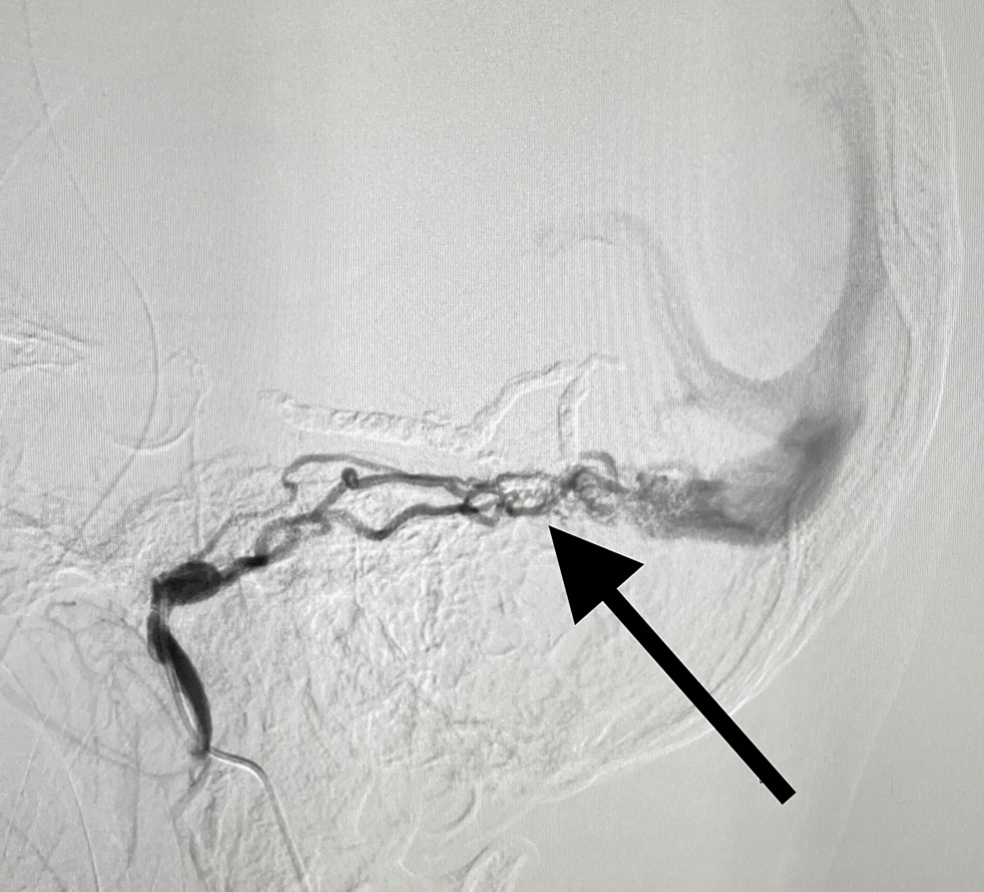
Cyanoacrylate glue embolization was done to close the abnormal arteriovenous connection, decrease the flow of blood between the aberrant arteriovenous system, and decrease the retrograde blood flow (black arrow)

Cyanoacrylate glue embolization was done to close the abnormal arteriovenous connection, decrease the flow of blood between the aberrant arteriovenous system, and decrease the retrograde blood flow, as shown in Figure 4.

The patient had a partial neurological recovery. He was discharged with active neuromusculoskeletal physiotherapy advice and did well after a two-month follow-up. Consent for the case report was obtained from the patient after ensuring he was fully informed about the details and purpose of the report and after addressing any questions or concerns he had.

## Discussion

In the dura mater of the brain, abnormal connections between veins and arteries are called DAVFs. While rare, these lesions can result in various neurological manifestations, ranging from mild symptoms to severe neurological deficits. Complex vascular lesions and DAVFs require a full understanding of their pathogenesis, diagnostic difficulties, and best practices [4].

DAVFs typically stem from organic origins. It is hypothesized that DAVFs involving brain veins frequently result from stenosis or total blockage of one of the brain’s dural venous sinuses, which are in charge of transferring blood from the brain to the heart. DAVFs may develop as a consequence of thrombosis in the dural venous sinuses [5]. This condition may give rise to heightened local venous pressure, leading to the development of abnormal arteriovenous shunts connecting the meningeal arteries and the dural venous sinuses, forming a pathological shunt.

The venous hypertension triggered by the obstruction of outflow can also restrict cerebral perfusion and cause neoangiogenesis [6]. Risk factors predisposing individuals to dural venous sinus thrombosis, including inherited prothrombotic conditions such as antithrombin, protein C and S deficiency, systemic illnesses, or conditions leading to prothrombotic states, along with trauma, are associated with the development of DAVF [3]. Timely detection and intervention are essential to prevent complications related to venous congestion and hemorrhage. Cerebral angiography has emerged as the preferred diagnostic method for identifying DAVFs, as it reveals the intricate network of abnormal artery-vein connections within the dura mater [7].

Various treatment options are used to manage the intracranial DAVF. These include a conservative approach, surgical intervention to close the shunt, an endovascular approach, and radiosurgical options. Radioablation, such as laser ablation of the abnormal connection and radioembolization where dyes are placed at the abnormal fistulous connection visualized on MR angiography and the glue, is applied to close the defect [8].

Due to the notable progress in neuroendovascular technology, open surgery is currently viewed as a secondary therapeutic option for DAVF patients. Increased obliteration rates may result from transarterial and transvenous embolization because certain DAVFs have an inaccessible lesion with a single pathway. Despite the remarkable advancements in endovascular embolization, certain patients still require surgery or radiosurgery to treat complex lesions [9].

A multidisciplinary strategy comprising neurologists, neurosurgeons, and interventional neuroradiologists is effective, as demonstrated by the positive outcome in this case. Collaboration among these specialties is imperative for optimal treatment planning and ensuring the best possible outcomes for patients. Regular post-embolization imaging and close clinical monitoring were essential for confirming the successful obliteration of the DAVF and tracking the patient’s symptomatic improvement over time [10].

## Conclusions

We treated a rare case of an arteriovenous fistula between the sigmoid and transverse sinus and the branches of the bilateral external carotid and right internal carotid artery using cyanoacrylate glue. This case report highlights the complexities involved in the diagnosis and management of DAVF. The successful outcome in this case underscores the importance of careful preoperative planning and collaboration among neurologists, neurosurgeons, and interventional neuroradiologists in addressing intricate DAVF.
